# Effect of Methylcellulose Molecular Weight on the Properties of Self-Assembling MC-g-PNtBAm Nanogels

**DOI:** 10.3390/bioengineering5020039

**Published:** 2018-05-23

**Authors:** Marion Jamard, Heather Sheardown

**Affiliations:** 1 School of Biomedical Engineering, McMaster University, Hamilton, ON L8S 4L8, Canada; marion.jamard@gmail.com; 2 Chemical Engineering, McMaster University, Hamilton, ON L8S 4L8, Canada

**Keywords:** methylcellulose, hydrophobization, molecular weight, self-assembly, drug delivery/release

## Abstract

The efficiency of drug delivery to the eye using topical drop therapy is limited by the ocular clearance mechanisms. Nanocarriers, able to encapsulate bioactive compounds and slow down their release, may allow for prolonged on-eye residence times when combined with topical application for treatment of ocular conditions. Previously, self-assemblies of methylcellulose (MC) hydrophobized with *N*-tert-butylacrylamide side chains (MC-g-PNtBAm) were developed. The purpose of the current study was to investigate the impact of the methylcellulose backbone length on the properties of the nanogels. We synthesized MC-g-PNtBAm nanogels using four different molecular weights of MC with two degrees of hydrophobic modification and investigated the physical and chemical properties of the resulting polymeric nanogels. While no significant change could be observed at a high degree of hydrophobization, properties were affected at a lower one. Increasing the molecular weight of MC improved the swelling capacity of the nanogels, increasing their size in water. An effect on the drug release was also noted. Nanogels prepared using MC with a molecular weight of 30 kDa did not retain as much dexamethasone and released it faster compared to those prepared using 230 kDa MC. Thus, besides the degree of hydrophobization, the length of MC chains provides another means of tuning the properties of MC-g-PNtBAm nanogels.

## 1. Introduction

Topical application of drugs is commonly used in the treatment of ophthalmic conditions [1]. These topical therapies have the advantages of simplicity, safety, and acceptance by patients. However, topical formulations have poor efficiency: in the eye, less than 5% of the drug reaches the intraocular tissue as a result of the effective clearance mechanisms and intrinsic biological barriers [2]. The barrier layers protect the eye from potentially harmful substances, including microorganisms and toxins, also prevent the entry and diffusion of drug molecules to the ocular tissues [3,4]. Rapid drainage through the nasolacrimal duct and constant dilution by the turnover of tears effectively limit the efficacy of topical formulations [5,6]. In order to achieve therapeutic efficacy, relatively high concentrations of drug and frequent administration are necessary. These bring an increased potential for side effects due to systemic uptake and a higher incidence of patient noncompliance [7,8,9]. Improving the bioavailability of drugs to achieve optimal drug concentration at the target site is thus one of the biggest challenges in ocular drug delivery. By treating ophthalmic diseases in a localized and efficient manner, systemic uptake of the drug is limited.

Current advances in the development of nanocarriers have shown that these systems have promise in ocular drug delivery [10,11]. Their small size enables better diffusivity across membranes and improved corneal permeability [4,11,12,13,14]. The high surface area-to-volume ratio may improve interaction with the ocular mucus membrane, leading to bioadhesion through interaction with the glycoproteins of the cornea and conjunctiva as well as mucopenetration [12,15]. This has the potential to minimize precorneal drug loss, particularly if the particles are prepared using or functionalized with a mucoadhesive material [12,16,17,18,19,20]. Such systems therefore have the potential to increase the efficiency of drug delivery by improving the bioavailability, while still allowing for the topical delivery of therapeutics.

We recently reported on the synthesis of self-assembling nanogels by grafting *N*-tert-butylacrylamide (NtBAm) hydrophobic side chains on methylcellulose (MC) to form MC-g-PNtBAm via cerium ammonium nitrate (CAN) [21]. MC-g-NtBAm amphiphilic molecules present the ability to organize themselves in aqueous media into a monodispersed and stable suspension of particles of a MC-based hydrophilic mesh, physically crosslinked by hydrophobic domains of PNtBAm [22]. The resulting nanogels presented an average diameter of 140 nm in water. Able to efficiently entrap dexamethasone in their hydrophobic domains, these particles presented promising results to overcome the limited solubility of hydrophobic drugs. They also showed the ability to provide controlled release of their payload, with a smaller burst phase and longer release compared to many previously reported nanocarriers [23,24,25,26]. Their self-assembling property presents the advantage of avoiding an extra step in the synthesis, sometimes involving harsh conditions potentially detrimental to the entrapped compound [27,28]. Varying the degree of NtBAm grafting with the feed concentrations of the monomer and initiator was found to affect the nanogel properties. Mucoadhesion through the MC functional groups was demonstrated. Higher degrees of hydrophobization (DH) resulted in nanogels with lower swelling capacity and more prolonged release. Incubation with human corneal epithelial cells demonstrated the potential biocompatibility of the nanogels.

Previous studies on hydrophobized polysaccharides have investigated the impact of the polysaccharide chain length on block-structured copolymers—that is, with the hydrophobic moiety attached to the end of the hydrophilic moiety [29,30,31]—and a few studies have investigated its effect as the backbone of a comb-structured copolymer [32]. In the present work, we investigate the impact of the methylcellulose backbone length on the nanogel properties. MC-g-PNtBAm nanogels were synthesized using four molecular weights of MC ranging from 30,000 kg/mol to 230,000 kg/mol at two different degrees of hydrophobic modification.

## 2. Materials and Methods

### 2.1. Materials

Methylcellulose (MC) Metholose SM-4, SM-25, SM-100, and SM-400 were purchased from Shin-Etsu (Totowa, NJ, USA). *N*-tert-butylacrylamide (NtBAm), cerium ammonium nitrate (CAN), and dimethyl sulfoxide-d6 (DMSO-d6) were purchased from Sigma-Aldrich (Oakville, ON, Canada), and dexamethasone from Sigma Life Science (D1756) (St. Louis, MO, USA). Phosphate buffered saline (PBS) 10 times concentrate was obtained from BioShop (McMaster University, Hamilton, ON, Canada). Nitric acid 70% was bought from EMD Chemical Inc. (Mississauga, ON, Canada).

### 2.2. Synthesis of MC-g-PNtBAm Nanogels

The synthesis of MC-g-PNtBAm nanogels was performed following a method previously described [21]. Briefly, 250 mg of MC of an appropriate molecular weight was dissolved in 50 mL water along with 200 mg of NtBAm. When dissolved, 0.5 mL of 70% nitric acid was incorporated into the solution. The mixture was purged with nitrogen for 30 min and either 50 mg or 250 mg of CAN dissolved in 1 mL of milliQ water was added to start the polymerization. The reaction was left stirring at room temperature for 24 h, followed by extensive dialysis (pre-wetted Regenerated Cellulose tubing 3.5 kDa, Spectrum Laboratories, Rancho Dominguez, CA, US) to remove any unreacted compound. The compositions of the different formulations are summarized in Table 1.

Various molecular weights of MC were used with two different CAN concentrations to synthesize the nanogels as shown in Table 1. Nanogel nomenclature is based on the molecular weight of MC and the degree of hydrophobization, calculated as the average number of NtBAm monomer for 100 anhydroglucose units (AGU) of the MC (determined by ^1^H NMR analysis [11]). For example, MC(165k)-g-PNtBAm_50% denotes a nanogel made with 165 kDa methylcellylose and 50 NtBAm units for 100 AGU.

### 2.3. ^1^H NMR Analysis

Freeze-dried materials were dissolved in DMSO-d6 and analyzed by ^1^H nuclear magnetic resonance (NMR, Bruker AVANCE 600 MHz NMR spectrometer).

### 2.4. Particle Size Measurements

Particle mean sizes were measured by single nanoparticle tracking using the Malvern NanoSight LM10.

### 2.5. Transmission Electron Microscopy Study

Samples were diluted 40-fold with purified water and 5 µL of the suspension was spread on 200 mesh Formvar-coated cooper grids without staining and allowed to dry under ambient atmospheric conditions. Transmission electron microscopy (TEM, JEOL 1200EX TEMSCAN) with 80 kV electron beam was used to view and photograph the morphology of the nanogels.

### 2.6. Loading of Dexamethasone

Dexamethasone was selected as a model ophthalmic drug for the release studies. To load dexamethasone into the nanogels, MC-g-PNtBAm synthesis was performed in a 0.01% w/v aqueous solution of dexamethasone, following the method described above. The suspension of loaded nanogels was then ultracentrifuged (Sorvall WX90) at 50,000 RPM for 30 °C at 23 °C. The amount of drug in the supernatant was measured by high-performance liquid chromatography (HPLC, Waters 2707 Autosampler, 1525 Binary HPLC Pump, 2489 UV/Visible detector, Column Dionex. Model Acclaim (r) 120 C18 5 μm 120A 4.6 × 250 mm) with a 254 nm detection wavelength, injecting 10 μL sample, and using a 1mL/min isocratic flow rate of 40:60 (v/v) acetonitrile/water. The following equation was used to calculate the loading efficiency of dexamethasone into the nanogels:

$$\begin{array}{c} Loadingoading\;efficiency\;efficiency\;(\%)\\ =100\\ \times \frac{(Initial\;amount\;oInitial\;amount\;of\;drugf\;drug-Amount\;of\;Amount\;of\;drug\;in\;supernatant\;drug\;in\;supernatant)\;}{InitialInitial\;amount\;of\;drug\;amount\;of\;drug} \end{array}$$

### 2.7. In Vitro Release of Dexamethasone

The in vitro release of dexamethasone from the nanogels was evaluated in phosphate buffered saline (PBS). 1 mL of a dispersion of drug-loaded particles (with a methylcellulose concentration of 4.5 mg/mL) in PBS was placed into a dialysis membrane (molecular weight cutoff 3500 Da, Spectra/Por, Spectrum Laboratories) which was immersed into 5 mL of PBS maintained at 32 ± 1 °C by a shaking water bath to approximately mimic on-eye conditions. Released dexamethasone was sampled by removing the release medium and replacing it with fresh prewarmed PBS at selected time points. The HPLC method described above was used to determine the concentrations of dexamethasone in the releasate. All measurements were performed in triplicate and plotted as the mean ± SD.

## 3. Results and Discussion

### 3.1. Synthesis

The influence of the molecular weight of the MC backbone on the nanogel properties was investigated using four different MC molecular weights (MW = 30,000 g/mol, 85,000 g/mol, 165,000 g/mol, and 230,000 g/mol). As the same mass was used for each, formulations all contained the same amount of anhydroglucose units (AGU). Two sets of materials were synthesized using two different initiator concentrations (1.82 × 10^−3^ mol/L and 9.12 × 10^−3^ mol/L: referred to as low and high initiation, respectively) while keeping constant NtBAm and nitric acid concentrations.

The amount of NtBAm grafted, referred to as the degree of hydrophobization (DH), was measured by ^1^H-NMR to look into the influence of the MC chain length. As in previous work [21,28,33,34,35], DH was deduced from the ratio of relative peak integrations of protons belonging to the hydrogen in the C2 position of MC and the CH_3_ group of NtBAm. The values are listed in Table 2. As previously observed [21], higher CAN concentrations resulted in higher grafting of NtBAm. When looking at the influence of MC MW, it appeared that for similar levels of initiator present in the mixture, similar DH were achieved for all molecular weights. All formulations presented a degree of hydrophobization of 30 ± 2% at lower levels of initiator and 49 ± 4% at higher levels of initiator, with no statistical difference (*p* = 0.1841). Results thus indicate that the length of the polysaccharide backbone did not impact the degree of NtBAm grafting. A previous study reported higher grafting levels for shorter polysaccharides, due to reduced steric hindrance exposing more reactive groups [32]. No such effect was observed here. The longer MC backbones possessed more PNtBAm side chains, but the overall hydrophobic/hydrophilic balance remained the same.

### 3.2. Impact of MC Molecular Weight on Particle Size and Morphology

**Figure 1:**
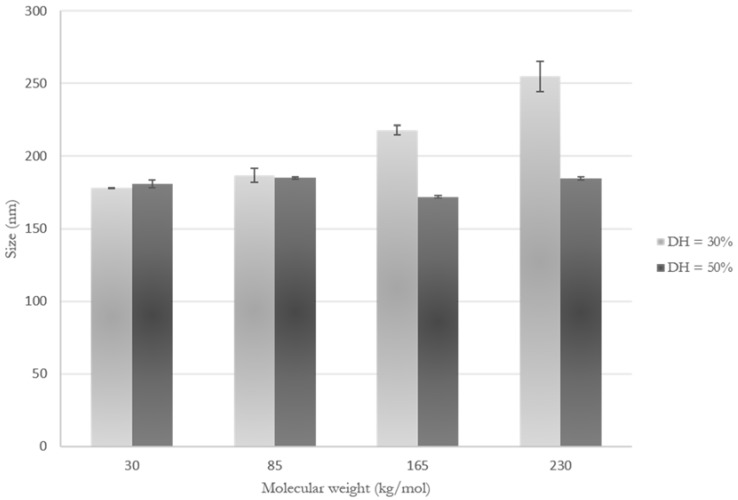
Hydrodynamic diameter of MC-g-PNtBAm nanogels varying the MC molecular weights at two different degrees of hydrophobization (DH), measured by particle tracking analysis. Three measurements were made, with the error bars corresponding to the standard deviation of the mean size.

At higher DH, the molecular weight of methylcellulose did not have an impact on the nanogel size in water, determined to be 180 nm ± 6 nm; while at a lower DH, increasing the molecular weight of MC significantly increased the size of the particles to up to 255 nm ± 10.5 nm (Figure 1). It has been previously shown that increasing DH decreased the swelling capacity of the nanogels [21,28,36]. It is thought that there is a higher frequency of hydrophobic chains along the polysaccharide backbone, induced by the higher feed ratio of initiator, supported by the fact that studies report that higher degrees of substitution result in nanogels of smaller sizes [35,37,38], while longer hydrophobic chains result in larger particles [31,34,39]. The high frequency of hydrophobic side chains tightens the aggregates and prevents the hydrophobic segments from expanding to take up water. The nanogels are thus not able to swell as much in aqueous media at high DH. At lower DH, however, the less numerous PNtBAm chains are separated by longer hydrophilic segments which are able to expand to take up water. The size of the assemblies thus increased with the MC molecular weight because of improved swelling capacity of the nanogels, in accordance with other studies [29,30,32].

**Figure 2:**
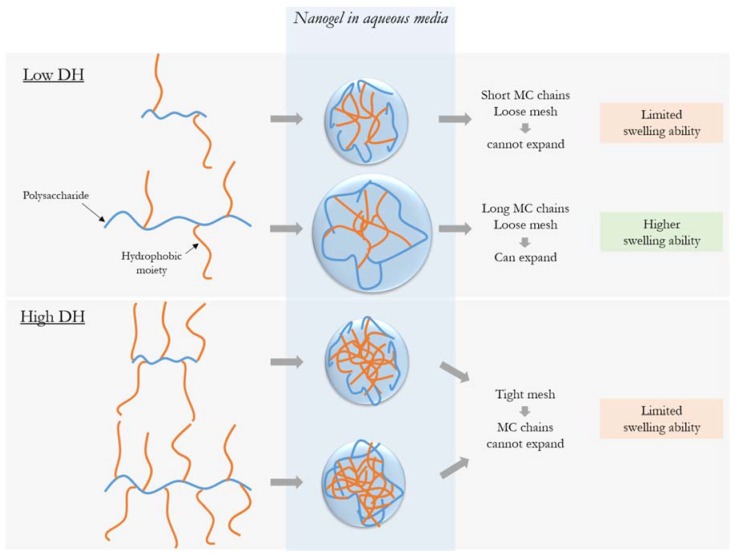
Scheme to illustrate the influence of MC molecular on the swelling ability of the nanogel (simplified with a single hydrophobic domain) at both degrees of hydrophobization. At high levels of hydrophobic modification, the tight mesh of the aggregates is unable to swell. Low levels of hydrophobic grafting create aggregates with a looser mesh, in which only longer MC chains can expand to take up water while maintaining the cohesion of the nanogel.

The impact of hydrophobization has been previously reported to depend on the size of the polysaccharide chain [29,32]. This is likely due to the ratio between the length of the hydrophobic side chains relative to the length of the hydrophilic segment between each side chain. When in water, longer hydrophilic MC chains are able to expand to take up and trap water while still maintaining the cohesion of the nanogel mesh, as illustrated in Figure 2; shorter chains do not create a mesh able to swell sufficiently to hold water molecules. However, at higher DH, the crosslinking density prevents volume change of the hydrophilic chains, making a mesh that is too tight to expand, so the molecular weight of MC no longer has an impact.

**Figure 3:**
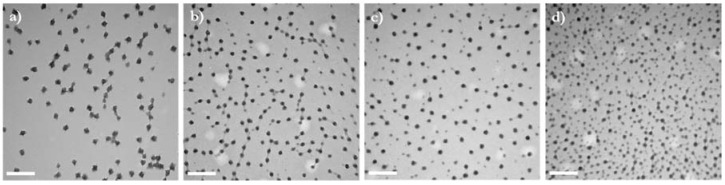
TEM pictures showing the morphology of nanogels with a DH of 30% and synthesized using various MC molecular weights: (**a**) 30 kg/mol, (**b**) 85 kg/mol, (**c**) 165 kg/mol, (**d**) 230 kg/mol. The scale bars indicate 500 nm.

TEM pictures showed that all formulations resulted in the formation of spherical particles (Figure 3). The diameters observed with TEM were smaller than those determined by Nanosight. This is in agreement with previous studies and can be attributed to the different states of samples during analysis [21,28,40,41,42]—dry and shrunken for TEM and suspended and swollen in water for Nanosight—as the high hydrophilicity of the polysaccharide increases the hydrodynamic diameter of the nanogels. TEM pictures reveal that particle size is less monodispersed when the MC length is increased. Such observations are in agreement with the statistics of the size measurement obtained with Nanosight and those previously made and attributed to the larger polydispersity of the polysaccharide at high MW [32]. The size of the nanogels in the dry state also seems to decrease when increasing the molecular weight of MC. The prevalence of intramolecular hydrophobic interactions due to the more numerous side chains along the polysaccharide backbone might result in aggregate formation with fewer copolymer molecules, which would explain their smaller size when dehydrated.

### 3.3. Impact of MC Molecular Weight on Drug Release

The impact of methylcellulose molecular weight on drug entrapment and release was studied using dexamethasone as a model hydrophobic and relevant ophthalmic drug. The highest and lowest methylcellulose chain lengths with both degrees of hydrophobic modification were selected for examination.

**Figure 4:**
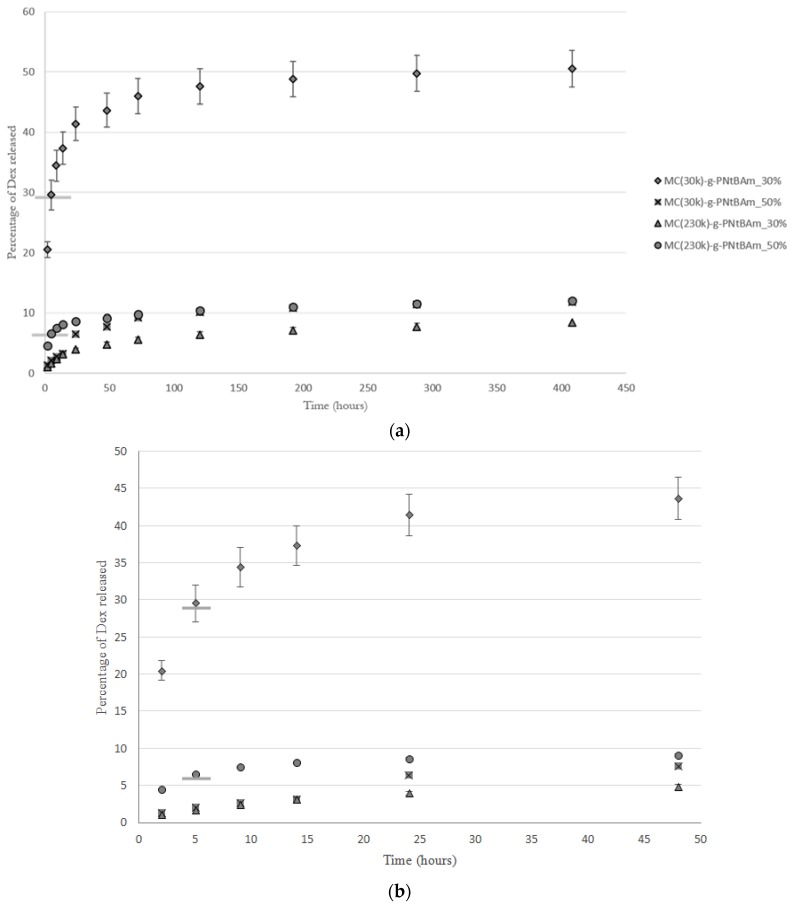
(**a**) Dexamethasone release from MC-g-PNtBAm nanogels at 30% or 50% DH and synthesized using MC of MW of 30 kg/mol or 230 kg/mol. The bars on the curves of MC(30k)-g-PNtBAm_30% and MC(230k)-g-PNtBAm_50% indicate the time point when the free drug in solution is released from the dialysis bag and the entrapped drug is being released. *n* = 3, with the error bars corresponding to standard deviations. (**b**) Magnification of the initial burst phase.

As previously observed [21], higher hydrophobic content induced higher loading efficiencies, due to stronger hydrophobic associations and/or the presence of increased hydrophobic domains (Table 3). The three-phase release observed was in accordance with the previous study on MC-g-PNtBAm nanogels [21] (Figure 4). An initial burst stage attributed to the drug soaked in the outer hydrophilic part of the aggregates is followed by a second slower diffusion phase corresponding to the dexamethasone that is more deeply entrapped. The final plateau stage is attributed to the high affinity of dexamethasone with the hydrophobic PNtBAm side chains [30].

At high levels of hydrophobic modification, both materials provided very high entrapment efficiencies and similar drug release rates. Those results indicate that molecular weight of methylcellulose had minimal impact on the entrapment and release properties of the nanogels at high DH. These observations are in accordance with the aggregate morphologies: the impact of MC molecular weight is impeded by the strong association into tight networks by the numerous hydrophobic chains.

However, at lower levels of hydrophobic modification, the nanogels prepared using the lower molecular weight MC entrapped significantly less dexamethasone than those prepared with the higher MC molecular weight and released it faster. Indeed, after the 30% of dexamethasone which was not entrapped came out of the dialysis bag, 20% of the payload in the nanogels was released over the next 15 days, compared to only up to 10% in all of the other gels. Small MC chains are thought to be less subjected to entanglement, and therefore more mobile and are not able to retain as much drug, letting the dexamethasone diffuse through more easily. On the contrary, longer chains are more efficient at entrapping the drug when forming nanogels, and formed stable entangled networks impeding drug diffusion. Interestingly, entrapment efficiencies were similar and seemed to be independent of DH for the longer MC chains. 

Noteworthy, while increasing the DH decreased the swelling capacity and the molecular weight of MC increased it, they both slowed the release of dexamethasone. Therefore, the diffusion rate of the drug seems to be controlled by the quantity of hydrophobic moieties and the mobility of the molecules forming the nanogel mesh. The length of the hydrophilic polysaccharide chain would determine the swelling ability and diffusion rate of the drug. Its impact on swelling is attributed to the ability of the longer MC chains to expand and take up water while remaining aggregated. In the same way, the openness of the mesh formed by shorter MC chains did not enable the uptake of water molecules; it also allowed for diffusion of the drug from the hydrophobic domains to the outside media. A comparison of the stability of the different nanogel formulations would be interesting to further investigate this analysis, as it has been reported that longer polysaccharide chains induced lower critical aggregation concentrations, thus giving better aggregate stability [32].

The release times, on the order of days, provide a substantial enhancement to the on-eye release times that are observed. Typically, less than 5% of the drug instilled via a typical eye drop would remain on the eye after 5 min. Therefore, penetration of the nanogels into the ocular mucin layer would allow for drug release to occur for periods of at least 3 to 4 days and potentially up to one week. Ongoing studies are focusing on examining the retention of the nanogels on the eye as well as the pharmacokinetics of drug release from the gels in the ocular environment.

## 4. Conclusions

When grafting hydrophobic side chains along the methylcellulose chain, the molecular weight of the polysaccharide backbone was found to only have an effect on the nanogel properties at low degree of hydrophobization. Indeed, while no significant change was observed at high DH, increasing the MC length created larger and more swollen particles at lower DH. In terms of drug release, shorter chains resulted in lower entrapment efficiencies and faster release of dexamethasone. Changing the molecular weight of MC thus provides an additional lever to tune the properties of MC-g-PNtBAm nanogels.

## Figures and Tables

**Table 1:** Molecular weight of MC and CAN concentration used for the synthesis of MC-g-PNtBAm formulations.

MC Molecular Weight (kg/mol)	30		85		165		230	
CAN concentration (mol/L)	1.82 × 10^−3^	9.12 × 10^−3^	1.82 × 10^−3^	9.12 × 10^−3^	1.82 × 10^−3^	9.12 × 10^−3^	1.82 × 10^−3^	9.12 × 10^−3^

MC, methylcellulose; CAN, cerium ammonium nitrate.

**Table 2:** Degree of hydrophobization (DH) for various MC molecular weights and initiator concentrations.

MC Molecular Weight (kg/mol)	30	85	165	230
Low initiation	28.00%	32.00%	32.80%	30.10%
High initiation	50.80%	50.20%	43.20%	51.60%

**Table 3:** Entrapment efficiencies of dexamethasone. *n* = 3, with error bars corresponding to the standard deviation.

MC Molecular Weight (kg/mol)	30	230
DH = 30%	71.61 ± 0.09%	98.96 ± 0.02%
DH = 50%	98.75 ± 0.09%	93.54 ± 0.03%
